# 

**Published:** 1887-10-15

**Authors:** 


### NOTICE TO ADVERTISERS.

THE scale of charges for small prepaid Advertisements—Situations Wanted, Situations Vacant, etc., etc., is as follows :—

| | s. | d. |
|---|---|---|
| One insertion of three lines | 1 | 0 |
| Three " " " | 2 | 6 |
| Per line afterwards | 0 | 6 |

*A line averages ten words.*

All copy for Advertisements must be sent to A. P. WATT, 2, PATERNOSTER SQUARE, E.C., by first post on the Tuesday *preceding* publication.

### NOTICE TO CORRESPONDENTS.

All correspondence must be written on one side of the paper only.

We cannot undertake to return MSS. not used.

**Communications,** whether intended for insertion or for private information, must be authenticated by the names and addresses of the writers, not necessarily for publication.

**Local papers** containing reports or news paragraphs **should be marked.**

It is especially requested that early intelligence of **local events of interest,** or which it may be thought desirable to bring under notice, may be sent direct to the Editor.

Communications respecting editorial matters should be addressed to the Editor, Norfolk House, Norfolk Street, Strand, London, W.C.

### AMUSEMENTS WITH PRIZES, FOR ALL AGES.

**Rules.**—All answers must be addressed to the PRIZE EDITOR, THE HOSPITAL, Norfolk House, Norfolk Street, Strand, W.C., not later than the *first* post on Friday next. The full name and address must in all cases be given, but a *nom de plume* may be added for publication. Only one side of the paper must be written on. No one will be permitted to win the Monthly or Quarterly Prizes twice in any one year, the annual prizes being offered especially to prevent this.

### FOR MEN AND WOMEN.

**Quarterly Prize Competition.**

Began 23rd July; ends 15th October, 1887.

**A Prize of Three Guineas**

will be awarded to the Competitor who shall have sent in the largest number of correct or best answers.

**A Prize of Five Guineas**

to the Competitor who shall have sent in the largest number of correct or best answers during the twelve months.

---

**No. 25. Double Acrostic.**

He was a genius of that way,
That upsets matter every day.
For the faculty with skill and care,
Valued prescriptions I prepare

---

1. If you should want a murder done,
Or anything to extremes run;
If you want folly, idiocy,
Do nothing more than send for me.

2. I'm a tree before the flame,
You burn me, yet I'm still the same.

3. I'm now a knave, and now a pet,
Though reprobate—a darling yet.

4. Beneath cathedral roof and wall
The sounds of *this* rise, swell, and fall.

5. In words remarkable for sense,
This statesman showed his eloquence:
He died, but England follows still
His struggle 'gainst "the People's Will."

6. Thou bird of omen ill, avaunt!
And cease the Southern Seas to haunt.

7. Fair swanlike beauty spread thy sails,
Thou "Sunbeam" whom the breeze ne'er fails.

**No. 26. Enigma.**

The highest gift of God to man,
When o'er his wondrous frame we scan.
What we sometimes lose in sorrow,
And often are compelled to borrow.
The lover's gift, the poet's song,
What art makes short
And nature long.

**Answers.**

No. 21. DOUBLE ACROSTIC.

| | | |
|---|---|---|
| I | nco | G |
| S | nuffe | R |
| A | yesh | A |
| A | zo | V |
| C | ure | I |
| N | augh | T |
| E | li | A |
| W | his | T |
| T | utt | I |
| O | trant | O |
| N | itroge | N |

Correct answers have been received from [illegible]rownie, Condorcet.

No. 22. MATHEMATICAL PUZZLE.

Let x=No.

a, b, c, d, the four parts.

$a+b+c+d=x$
$a+b=d$ $\therefore d=\frac{1}{2}x$
$a+2=b-2$
$\therefore 2d+c=x$ $\therefore c={}^1x$
$\therefore b-a=4$
Also $4c=d$ $\therefore a+b=\frac{1}{2}x$

[illegible]ollows that the No. is any multiple of 9.

The 4th part is always $\frac{4}{9}$ of No.

.. 3rd „ „ $\frac{1}{9}$ „

+ difference of 2nd from 1st=4.

[illegible]nswers have been received from Ellice, [illegible]ownie, Gretta, May, K. M., Arale, Condorcet, Esau, Mater, Perseverance.

### FOR CONVALESCENTS.

IN the many excellent hospitals existent in this country the sick are now provided with all that can be needed to promote their speedy convalescence. When, however, the s age is reached where disease has ceased and health has to be restored, then the active benevolence of those able to help is sometimes relaxed. The absence or presence of aids to cheerfulness, which in the sick are often the highroad to recovery, makes convalescence one of the mo[illegible] trying and critical, as it is certainly one of the p[illegible]easantest or most wearisome periods of a lon[illegible] illness. Anything which can promote cheerfulness, then, would certainly commend itself to those who have the interests of the sick at heart.

Amusements must generally be chosen with regard to their suitability for the condition of each patient, but there are amusements which can be utilised upon a larger scale, with the view of making an impression on the minds of the convalescents, and so to afford them a subject for conversation and thought. To this latter class belong the concerts, dramatic performances, and evening entertainments of various kinds, which are now given during the winter months by amateurs and other friends of hospitals. When such a scheme is found to be beyond the means or requirements of any institution, we should strongly recommend recitations and readings, which if less pretentious than the theatricals, will possibly be found quite as amusing and instructive. Good readers who can read aloud, and who know how to select suitable pieces are frequently a great addition to the family circle in the winter evenings, and would prove a real boon in many a hospital and asylum Dickens's works, the Irish scenes so cleverly depicted by Lever and many other sparkling writers, and the clever and entertaining Ingoldsby Legends, are the class of subjects best adapted to such a purpose.

We heartily commend this idea of a general entertainment to the managers and directors of all institutions where the amusement of convalescents is not at present attempted. The entertainments need not be too frequent, but they undoubtedly create a joyful anticipation in the minds of most patients, and very often leave exceedingly pleasant recollections. Reading, good reading, to a number of people, creates more impression than reading to an individual, for the minds of the various listeners are pleasantly excited by the companionship of others, and may be thus introduced to some of our choicest authors, with whom otherwise they might never become acquainted.

Many patients find great entertainment in reading for themselves, and every hospital—in fact, every ward—should possess a library of suitable works, in the selection of which the patients' own wishes shou'd be consulted, though a careful and shrewd guidance of that choice may be exercised. There will be many patients, however, who for various reasons, will be unable to read in this way; and for some of these, draughts, dominoes, and sometimes chess may b[illegible] provided with advantage. There are now so many indoor games, that the choice can be left to the sister or nurse. Besides these games, nurses should be encouraged to impart to their patients such knowledge as many of them possess in the more technical branches of sewing, working wool, basket-making, etc. The fingers may often be employed when the mind is much too weary, and patients may be easily instructed to make mats woollen balls, and many other pretty and useful articles on the kindergarten system, of which a list can be obtained on application to the Kindergarten Emporium, Miss E. Frost, Manageress, 57, Berners Street, London, W.

We should be happy to receive practical suggestions, and an account of the way in which the winter evenings have been spent at any hospital, asylum, or kindred institution here, in the Colonies, or in America. It is the aim of THE HOSPITAL to be useful, as well as interesting, and some patients and many officials, sisters, nurses, and hospital visitors might find relaxation in wri[illegible]ing an account of how they have whiled away the hours of convalescence, and so promoted health and secured amusement with profit.

### THE CHILDREN'S CORNER.

PRIZES.

FOR MOST CORRECT ANSWERS TO PUZZLES.
One Pound per Month.

FOR BEST LITTLE LETTERS.
Ten Shillings per Quarter.

A PRIZE OF THREE GUINEAS
to the Competitor who shall have sent in the largest number of correct or best answers during the twelve months.

---

This week I welcome with much pleasure many new competitors and some of my old friends, who, after a quarter's retirement, have re-entered for the competitions, to which they look forward as a pleasant pastime for the winter evenings.

**Puzzles—Third Week.**

No. 9. NATURAL HISTORY ACROSTIC. 1. A caterpillar. 2. A species of rat. 3. A small carnivorous animal. 4. A waterfowl. 5. A small animal noted for its skin. 6. A genus of fishes. Initials and finals give the name of a small American animal.

No. 10.
A monarch, a letter, and a weight combined,
The name of a town in England you'll find.

No. 11. A troublesome insect, if transposed, forms an animal's feet; take away one letter, and you find the past tense of a verb, which, if reversed, gives a useful instrument.

**Letters—Third Week.**

For the weekly letters during the winter, I shall take the principal *Castles* and *Cathedrals* of Great Britain, and I want you each to tell me in your own words the chief events of historical interest connected with them. We cannot do better than begin this week with "Westminster Abbey."

**Results of First Week—Puzzles.**

No. 1. HISTORICAL CHARADE. Holy-rood = Holyrood.

No. 2. FLORAL ANAGRAMS. Anemone, Clematis, China Rose, Acanthus.

No. 3. RIDDLE-ME-REE. Orange.

No. 4. CONUNDRUM. The poet who offers a refuge to lions is Dryden.

*All right*—Punch, Judy, Toby, Paddy, Spider, Jolly Sailor, Poodle, Pepita, Tim, Ossian, Skye, A. G. S. McCulloch, Mount Edgcumbe, A. C. K., Alsie, Gem, Jerusalem, Paul Methven, R. E. Herbertson, Scrooge, Uncas, G. B. P., Joe; *Three right* Nurse E., Ellie, Fern; *Two right*—Princess Ida.

**Letters.**

The letters this week on "Your Favourite Hero or Heroine in History," are on the whole well and thoughtfully written. Examples have been taken from ancient and modern history. Twaddle, amongst other remarks, writes of his favourite hero, Socrates:—

I think that my favourite character in all history, is the great heathen philosopher, Socrates. He was so noble in condemning injustice, in sticking up for ill-used criminals in the teeth of public opposition He, too, was the first to see a higher divinity and a higher religion than the belief in the heathen gods. Public opinion he did not care for, and that is chiefly to be admired in him Like Pluto and the Roman Cicero, he steadfastly believed in the immortality of the soul

Princess Ida chooses Charles I as her hero, and sends an interesting letter touching on the chief events in the reign and life of this unfortunate king. Among the other heroes and heroines selected are: Richard I, Joan of Arc, Queen Philippa, Prince Rupert, Nelson, Wellington, etc. Pressure on space prevents my remarking further on the various letters received.

---

Three marks each:—Poodle, Princess Ida, Twaddle, Mount Edgcumbe, Skye, Punch. Two marks each—Ossian, Gem, Pepita, Fern.

### Hospitals, Doctors, Treatment, and Attendance.

### THE IN- AND OUT-PATIENTS' HOSPITAL DIARY.

This Diary is so arranged as to make some portion of it appear every week, and the whole in each monthly part. It has been very difficult to compile, and corrections will at all times be gratefully received. It has been suggested that similar information about the larger Provincial and Scotch Hospitals would be useful to many readers. We should be glad to hear if this is felt to be the case.

### GENERAL HOSPITALS—*continued.*

| Hospitals and Terms of Admission. | Physicians, Surgeons, and Medical Officers, with Days and Hours of Attendance. |
|---|---|
| **University College Hospital** (or **North London**), Gower Street, W.C. Sec., Mr. N. H. Nixon. Accidents and emergencies free. All other cases by Governor's letter of recommendation | *In-Physicians*, Drs. W. Fox, Tu. Th. 2, S. 9.30; Ringer, M. W. F. 1.30; Bastian, M. 2.30, W. 10, F. 2.30; F. Roberts, Tu. 1.15, Th. 1.30, S. 9.30. *In-Surgeons*, Messrs. B. Hill, Tu. 2.15, W. 2, F. 2.15; C. Heath, M. W. F. 2; M. Beck, Tu. Th. S. 2. *Out-Physicians*, Drs. Gowers, W. S.; Poore, Tu. F.; Barlow, M. Th. Hour, 1.30. *Out-Surgeons*, Messrs. Barker, M. Th.; Godlee, Tu. F.; Horsley, W. S. Hour, 1.30 |
| **West London Hospital**, Hammersmith Road, W. Sec. and Supt., Mr. R. J. Gilbert. Accidents and emergencies free at all times. Other cases require a letter of recommendation. New cases must attend at 1.30 | *In-Physicians*, Drs. G. Rogers, D. W. Hood, F. Drewitt. *In-Surgeons*, Messrs. C. Keetley, F. Edwards, W. B. Clarke. *Out-Physicians*, Drs. Herringham, M. Th.; Ball, Tu. F.; S. Taylor, W. S. Hour, 2. *Out-Surgeons*, Messrs. Ballance, Tu. F.; Weiss, M. Th.; Wainewright, W. S. Hour, 2 |
| **Westminster Hospital**, Broad Sanctuary, S.W. Sec., Mr. S. M. Quennell. Accidents and urgent cases free at all times. Other cases require a letter of recommendation | *In-Physicians*, Drs. Sturges, M. Th. S.; Allchin, M. Tu. F.; Donkin, Tu. W. S. Hour, 1.30. *In-Surgeons*, Messrs. Cowell, M. Tu. Th.; Davy, M. Tu. F.; Macnamara, M. W. S. Hour, 1.30. *Out-Physicians*, Drs. De H. Hall, M. Th.; A. H. Bennett, Tu. F.; Murrell, W. S. Hour, 1.30. *Out-Surgeons*, Messrs. Cooke, M. Th.; Bond, Tu. F.; Barrow, W. S. Hour, 1.30 |

### II.—SPECIAL HOSPITALS.

#### CANCER.

| | |
|---|---|
| **Cancer Hospital**, Brompton, S.W. Sec., Mr. W. H. Hughes. Entirely free to both in- and out-patients | *In- and Out-Surgeons*, Drs. A. Marsden, W.; H. Snow, M.; F. A. Purcell, F.; Messrs. F. B. Jessett, W.; C. Stonham, Th.; C. Jennings, Tu.; W. H. Elam, S. Hour, 2 |
| London Hospital, Whitechapel Road, E. | Daily, 1.30. *See* General Hospitals |
| Middlesex Hospital, Berners Street, W. Admission free | *In-Surgeons*, Messrs. Hulke, Lawson, and Morris. *Out-Surgeon*, Mr. Morris, Th. 1.30 |
| **St. Saviour's Hospital**, Osnaburg Street, N.W. Sec., Mr. E. Poitiers. By Governors' letters | *In- and Out-Physician*, Dr. A. Kennedy, 10.30 and 4 daily |

#### CHILDREN'S DISEASES.

| | |
|---|---|
| **Alexander Hospital for Children**, 18, Queen Sq., W.C. Sec., Mrs. Marsh. For hip disease | *Physician*, Dr. Steavenson. *Surgeons*, Messrs. H. Marsh, Tu. F.; A. Bowlby, M. Th. Hour, 4.30 (No out-patients) |
| **All Saints' Children's Hosp.**, 4, Margaret St., W. For chronic joint diseases | *Surgeon*, Mr. N. Smith (No out-patients) |
| **Belgrave Hosp. for Children**, 77 & 79 Gloucester St., Pimlico. Hon. Secs., Capt. Stopford, and Rev. J. Storrs. By letters; accidents free | *In- and Out-Physicians*, Drs. W. Hope and W. Ewart, M. Th. 9. *In- and Out-Surgeons*, Messrs. C. T. Dent and Margerison, Tu. F. 9 |
| **Charing Cross Hospital**, West Strand, W.C. | *Out-Physician*, Dr. Nursby, W. S. 1.30 |
| **Cheyne Hospital for Sick and Incurable Children**, 46, Cheyne Walk, Chelsea, S.W. Sec., Miss D. Evans | *Physician*, Dr. G. V. Poore. *Surgeons*, Messrs. T. P. Bartlett and J. Macready attend alternate days in afternoons. (No out-patients) |
| **East London Hospital for Children and Dispensary for Women**, Glamis Road, Shadwell, E. Sec., Mr. Ashton Warner. By Governor's letter. Urgent cases and accidents are admitted free | *In-Physicians*, Drs. Donkin, M. Th.; Eustace Smith, T. F.; Crocker, F. *In-Surgeons*, Messrs. A. Cæsar, R. W. Parker, W. F. *Out-Physicians*, Drs. Crocker, M.; Dawson Williams, Tu. Th.; Coutts, W. F. Hours, 1 and 3. *Out-Surgeon*, Mr. Dunn, Tu. F. 1, 3 |
| **Evelina Hospital for Sick Children**, Southwark Bridge Road, S.E. Sec., Mr. T. S. Chapman. Free, but Governor's letter takes precedence. In-patients (boys) between 2 and 10, (girls) between 2 and 12 | *In-Physicians*, Drs. F. Taylor and J. Goodhart. *In-Surgeons*, Messrs. H. G. Howse and R. C. Lucas. *Out-Physicians*, Drs. N. Tirard, M. Th. 9; F. Willcocks, Tu. F. 9. *Out-Surgeons*, Messrs. C. J. Symonds, S. 9; G. H. Makins, W. 9 |

#### CHILDREN'S DISEASES—*continued.*

| Hospitals and Terms of Admission. | Physicians, Surgeons, and Medical Officers, with Days and Hours of Attendance. |
|---|---|
| **German Hospital**, Dalston Lane, Dalston, E. | *In-Physician*, Dr. Rasch, M. Th. 9 |
| **Great Northern Central Hosp.** Caledonian Rd., N. | *Physicians*, Drs. Murray and Barnes, W. 2.30 |
| **Grosvenor Hospital for Women and Children**, Vincent Sq., S.W. Hon. Secs., Mr. A. J. Ram and Miss Day. By letter or payment | *In- and Out-Physicians*, Drs. R. Gibbons and Steavenson, Tu. W. Th. S. 2. *In- and Out-Surgeons*, Messrs. Smythe and Parker, Tu. W. Th. S. 2 |
| **Hospital for Sick Children**, 48 and 49, Great Ormond St., W.C. Sec., Mr. S. Whitford. By Governors' letters. If without letter, the case of the applicant is investigated. In-patients between 2 and 12; Out-patients under 12 | *In-Physicians*, Drs. W. Cheadle, Tu. F.; Sturges, W. S.; Barlow, M. Th. Hours, 9 to 11. *In-Surgeons*, Messrs. H. Marsh, W. S.; E. Owen, W. S. Hours, 9 to 11. *Out-Physicians*, Drs. R. J. Lee and Abercrombie, M. Th.; Lubbock and Money, Tu. F. Hours, 8.30 to 10. *Out-Surgeons*, Messrs. Morgan and Pitts, W. S. 8.30 to 10 |
| **King's College Hospital**, Portugal Street, W.C. | *In- and Out-Physician*, Dr. T. C. Hayes, W. F. 1 |
| **Middlesex Hospital**, Berners Street, W. Free. Children under 3 | *Out-Physician*, Dr. Duncan, W. S. 1.30 |
| **North Eastern Hospital for Children**, Hackney Road, E. Sec., Mr. A. Nixon. By free ticket or small payment | *In- and Out-Physicians*, Drs. W. Cayley, F. Turner, C. Semple, and W. Pasteur, M. W. Th. S. 1.30. *In- and Out-Surgeons*, Messrs. Waren Tay and Pollard, M. 1.30, F. 9 |
| **Paddington Green Children's Hosp.**, Paddington Green. Sec., Mr. W. H. Pearce. Admission free; boys under 12; girls under 14 | *In- and Out-Physicians*, Drs. T. Lauder Brunton, Ogilvie, Tu. F.; G. Laycock, M. Th.; S. Phillips, W. S. Hour, 9.15. *In- and Out-Surgeons*, Messrs. W. W. Cheyne, M., S. Boyd, F. Hour, 9.15 |
| **Royal Hospital for Children and Women**, Waterloo Bridge Road, S.E. Sec., Mr. R. G. Kestin. Out-Patients pay 1d. on each attendance. Boys not admissible, as out-patients, over 12, or, as in-patients, over 8 | *In-Physicians*, Drs. Park, M. Th.; Gulliver, Tu. F.; Price, W. S. Hour, 2. *In-Surgeon*, Mr. W. H. A. Jacobson, M. Th. Hour, 2. *Out-Physicians*, Drs. Park, M. Th.; Dakin, W. F.; Hadden, Tu. S. Hour, 2. *Out-Surgeon*, Mr. E. O. Day, W. 2 |
| **St. Mary's Hospital**, Cambridge Place, Paddington | *Out-Physician*, Dr. M. Handfield Jones, M. Th. 1.30 |
| **St. Thomas's Hosp.**, Westminster Bridge Road, S.E | *Out-Physician*, Dr. Cory, W. S. 1.30 |
| **Samaritan Free Hosp. for Women and Children**, Lower Seymour Street, W.; Branch, 1, Dorset St., W. Sec., Mr. G. Scudamore. In-patients must be between 2 and 12. Out-patients Department (free) is at Edward's Mews, Duke Street, W. | *In-Physician*, Dr. W. H. Day. Daily, 2. *In-Surgeon*, Mr. W. A. Meredith. Daily, 2. *Out-Physicians*, Drs. M. Prickett and Amand Routh, W. S. 1.30. *Out-Surgeons*, Messrs. W. A. Meredith and J. D. Malcolm, M. Th.; A. H. G. Doran and W. S. A. Griffith, Tu. F., 1.30 |
| **Victoria Hosp. for Children**, Queen's rd., Chelsea, S.W. Sec., Capt. W. C. Blount, R.N. Age of boys, 2 to 12 years; age of girls, 2 to 16. Admission for in-patients by subscriber's letter. Accidents and urgent cases free at all times | *In-Physicians*, Drs. J. Evans, M. Th. 2; Ridge-Jones, Tu. F. 2. *In-Surgeons*, Messrs. Pick, Tu. F. 9.15; Clutton, M. Th. 4.30. *Out-Physicians*, Drs. A. Venn, W. S. 9.30; C. Fox, M. Th. 1.30; F. D. Drewitt, Tu. F. 9; J. Philpot, M. Th. 9. *Out-Surgeons*, Messrs. F. Churchill, Tu. F. 10; W. Pye, M. Th. 9.30 |
| **West London Hospital**, Hammersmith rd, W. | Daily, 2. *See* General Hospitals |

#### CONSUMPTION AND DISEASES OF THE CHEST.

| | |
|---|---|
| **City of London Hosp. for Diseases of the Chest**, Victoria Park, E. Sec., Mr. T. Storrar Smith. By subscriber's letter available for 6 weeks. | *In-Physicians*, Drs. J. Thorowgood, S.; E. Smith, M.; J. Fothergill, W.; S. West, F.; G. A. Heron, W.; V. D. Harris, Tu. Hour, 2. *Out-Physicians*, Drs. V. D. Harris, Tu.; J. A. Ormerod, Th.; E. C. Beale, Tu. F.; B. Fenwick, W. S.; A. Money, W. S.; L. E. Shaw, M. Th. Hour, 2 |
| **Hospital for Consumption**, Fulham rd., Brompton, S.W. Sec., Mr. H. Dobbin. Admission by letter of recommendation, but, where unobtainable, application may be made to the Secretary at the Hospital. | *In-Physicians*, Drs. F. S. Thompson, M. Th.; C. T. Williams, M. Th.; R. D. Powell, Tu. F.; J. Tatham, W. S.; R. E. Thompson, W. S.; F. T. Roberts, Tu. F. Hours, 2 to 4. *In-Surgeon*, Mr. R. J. Godlee, F. *Out-Physicians*, Drs. T. H. Green, J. M. Bruce, M. Th.; J. K. Fowler, W. S.; P. Kidd and C. Y. Biss, Tu. F.; T. D. Acland, W. S. Hour, 12 |

### Hospitals, Doctors, Treatment, and Attendance.

### THE IN- AND OUT-PATIENTS' HOSPITAL DIARY.

This Diary is so arranged as to make some portion of it appear every week, and the whole in each monthly part. It has been very difficult to compile, and corrections will at all times be gratefully received. It has been suggested that similar information about the larger Provincial and Scotch Hospitals would be useful to many readers. We should be glad to hear if this is felt to be the case.

### CONSUMPTION AND DISEASES OF THE CHEST.—*Continued.*

| Hospitals and Terms of Admission. | Physicians, Surgeons, and Medical Officers, with Days and Hours of Attendance. |
|---|---|
| **North London Hospital for Consumption and Diseases of the Chest,** Mount Vernon, Hampstead, N. Sec., Mr. L. F. Hill. Admission by subscriber's letter, available for 6 weeks. | *In-Physicians*, Drs. A. Evershed, Tu.; B. O'Connor, F.; D. Pidcock, F. Hour, 10.30. *Out-Physicians*, Drs. B. O'Connor, D. Pidcock, J. E. Squire. Daily, 2 (attend at Out-patients' Branch, 216, Tottenham Court Road, W.) |
| **Royal Hospital for Diseases of the Chest,** 231, City Road, E.C. Sec., Mr. John J. Austin. By Governor's letter, available for six weeks | *In-Physicians*, Drs. Hensley, W.; Gilbart-Smith, M.; Finlay, Th. *In-Surgeon*, Mr. A. Pearce-Gould, M. Tu. W. Th. F. 2, S. 9. *Out-Physicians*, Drs. White, Tu. F.; Pringle, M. Th.; O. Browne, W. S.; A. Davies, M. F.; H. Habershon, W. S.; E. Stewart, Tu. Th. Hour, 1; S. 9 |
| **Royal National Hosp. for Consumption and Diseases of the Chest,** Ventnor. Offices, 34, Craven St., Strand, W.C. Sec., Mr. E. Morgan. Admits only incipient cases by Governor's letter. | *In- and Out-Physicians*, Drs. J. G. Sinclair Coghill, and R. Robertson attend daily (at Ventnor) on out-patients at 11; on in-patients at noon. *In- and Out-Surgeons*, Messrs. Whitehead, Williamson |

### EAR CASES.

| Hospitals and Terms of Admission. | Physicians, Surgeons, and Medical Officers, with Days and Hours of Attendance. |
|---|---|
| **Central London Throat and Ear Hosp.,** Gray's Inn Rd., W.C. Sec., Mr. R. Kershaw. Free, but when able, a small payment is expected | *In- and Out-Surgeons*, Mr. Lennox Browne, M, Th. 2.30; Dr. Orwin, W. S. 2.30; Dr. Grant, Tu. 5, Th. 2.30; Messrs. P. S. Jakins, M. W. 2.30; F. 5; T. Carmalt Jones, M. Th. S. 2.30 |
| **Charing Cross Hospital,** West Strand, W.C. | *In- and Out-Surgeon*, Mr. Cantlie, F. 9 |
| **Great Northern Central Hospital,** Caledonian Road, N. | *In- and Out-Surgeon*, Mr. A. E. Cumberbatch, W. 9 |
| **Guy's Hospital,** St. Thomas's Street, S.E. | *In- and Out-Surgeon*, Mr. Purves, Tu. F. 12.30 |
| **Hospital for Diseases of the Throat,** Golden Sq., W. Sec., Mr. W. T. Sharp | *Physicians*, Drs. Wolfenden, Tu. F. 2.30; Macdonald, Tu F. 6.30; Bond, W. S. 2.30. *Surgeon*, Mr. T. M. Hovell, M. Th. 2.30 |
| **King's College Hospital** Portugal Street, W.C. | *In- and Out- Surgeon*, Mr. Pritchard, Th. 2.30 |
| **London Hospital,** Whitechapel Road, E. | *In- and Out-Surgeons*, Dr. Woakes, Mr. T M. Hovell, S. 9 |
| **Middlesex Hospital,** Berners Street, W. Free. | *Out-Surgeon*, Mr. Hensman, Tu. 9 |
| **National Hosp. for the Paralysed, etc.,** Queen Sq., W.C. | *In- and Out-Surgeon*, Mr. A. E. Cumberbatch, W. 2 |
| **North West London Hosp.,** 18, Kentish TownRd., | *Out-Surgeon*, Mr. Collier, M. 1.30 |
| **St. Bartholomew's Hospital,** Smithfield, E.C. | *Surgeon*, Mr. Cumberbatch, Tu. F. 2 |
| **St. George's Hospital,** Hyde Park Corner, S.W. | *In- and Out-Surgeon*, Sir W. B. Dalby Tu. 1.30 |
| **St. Mary's Hospital,** Cambridge Place, Paddington, W | *In- and Out-Surgeon*, Mr. G. P. Field, M. Thur. 3 |
| **St. Thomas's Hospital,** Westminster Bridge Rd., S.E. | *Out-Surgeon*, Mr. Clutton, M. 1.30 |
| **University College Hospital,** Gower Street, W.C. | *In- and Out-Surgeon*, Mr. Barker, Th. 9 |
| **Westminster Hospital,** Broad Sanctuary, S.W. | *In- and Out-Surgeon* Mr. Barrow, Tu. F. 9 |

### EYE DISEASES.

| Hospitals and Terms of Admission. | Physicians, Surgeons, and Medical Officers, with Days and Hours of Attendance. |
|---|---|
| **Central London Ophthalmic Hospital,** 238a, Gray's Inn Rd., W.C. Sec., Mr. G. H. Leah. Admission free | *Surgeons*, Messrs. T. Archer, G. Abbott. W. Charnley, W. Jesson. E. Clarke, J. R. Kemp and Granville Barker (House Surgeon). Daily, 1 |
| **Evelina Hospital for Sick Children,** Southwark Bridge Rd. | *Surgeon*, Dr. W. Brailey, Th. 1 |
| **Great Northern Central Hosp.,** Caledonian Rd., N. | *Surgeon*, Mr. Hutchinson, Jnr., Tu. F. 9.30 |
| **Guy's Hospital,** St. Thomas's Street, S.E. | *Surgeons*, Mr. Higgens, Dr. Brailey, Tu. F. 12.30 |
| **Hospital for Sick Children,** 49, Gt. Ormond St., W.C. | *Surgeon*, Mr. R. Marcus Gunn, Tu. 2 |
| **King's College Hosp.,** Portugal Street, W.C. | *Surgeon*, Mr. McHardy, M. W. Th. 1.30 |
| **London Hospital,** Whitechapel Road, E. | *Surgeons*, Messrs. Waren Tay, S. 9; Eve, Tu. 9 |
| **Middlesex Hospital,** Berners Street, W. | *Surgeon*, Mr. Lang, Tu. F. 9 |

### EYE DISEASES.—*Continued.*

| Hospitals and Terms of Admission. | Physicians, Surgeons, and Medical Officers, with Days and Hours of Attendance. |
|---|---|
| **National Hospital for the Paralysed, etc.,** Queen Sq., W.C. | *Surgeons*, Messrs. R. Brudenell Carter, F. 4; R. Marcus Gunn, Tu. 4 |
| **North-West London Hospital,** 18, Kentish Town Rd., N.W. | *Surgeon*, Dr. W. Collins, F. 3.30 |
| **Paddington Green Children's Hosp.,** Paddington Green | *Surgeon*, Mr. W. H. H. Jessop, Th. 9 |
| **Royal Free Hospital,** Gray's Inn Road, W.C. | *Surgeon*, Mr. Mackinlay, M. F. 9 |
| **Royal London Ophthalmic Hospital,** Blomfield Street, Moorfields, E.C. Sec., Mr. R. J. Newstead. Admission free to the poor. | *Surgeons*, Messrs. J. W. Hulke, W. S.; G. Lawson, Tu. F.; J. Couper, Tu. F.; W. Tay, M. Th.; J. Tweedy, Tu. F.; E. Nettleship, W. S.; R. M. Gunn, W. S.; Lang, M. Th. Hours, 8 to 10 |
| **Royal South London Ophthalmic Hosp.,** 6, St. George's Circus, S.E. Sec., Mr. C. Comyn. Free to the necessitous poor; or by payment | *Surgeons*, Messrs. Watson, McHardy. Daily, 2 |
| **Royal Westminster Ophthalmic Hospital,** King William Street, W.C. Sec., Mr. T. Beattie-Campbell. Poor and accidents free | *In- and Out-Surgeons*, Messrs. Power, M. F.; Frost, M. Th.; Rouse, Tu. S.; Hartridge, Tu. F., Macnamara, M. Th.; Cowell, W. S.; Juler, W. S. Hour, 1 |
| **St. Bartholomew's Hospital,** Smithfield, E.C. | *Surgeons*, Messrs. Power, Th. 2; Vernon, Tu. S. 2.30 |
| **St. George's Hospital,** Hyde Park Corner, S.W. | *Surgeons*, Messrs. B. Carter, Frost, M. Th. 2; F. 1.15; W. S. 2 |
| **St. Mary's Hospital,** Cambridge Place, Paddington. W. | *Surgeons*, Messrs. A. Critchett, H. Juler, Tu. F. S. 9 |
| **St. Thomas's Hospital,** Westminster Bridge Rd., S.E. | *Surgeons*, Messrs. Nettleship, Tu. Th. F. 1.30; Lawford, M. W. 1.30 |
| **University College Hospital,** Gower Street, W.C. | *Surgeon*, Mr. Tweedy, M. Tu. Th. F. 2 |
| **West London Hospital,** Hammersmith Road, W. | *Surgeons*, Messrs. B. Vernon, H. Dunn, Tu. Th. S., 2 |
| **Westminster Hospital,** Broad Sanctuary, S.W. | *Surgeon*, Mr. Cowell, M. Th. 2 |
| **Western Ophthalmic Hospital,** 153 and 155, Marylebone Road, W. Sec., Mr. G. E. Manton. By letter or small payments; free to poor | *Surgeons*, Drs. Miller M.; Collins, F.; Wheatly, M. Th.; Messrs. Carmal. Jones, W.; Buxton, Tu. F.; Nunn, W. S. Hour, 2 |

### ORTHOPÆDIC CASES.

| Hospitals and Terms of Admission. | Physicians, Surgeons, and Medical Officers, with Days and Hours of Attendance. |
|---|---|
| **City Orthopædic Hosp.,** 27, Hatton Garden, E.C. Sec., Mr. E. Dereuth. Admission free | *Surgeons*, Mr. E. J. Chance and House-Surgeon. M. Tu. Th. F. 2.30. New cases Tu. F. 2.30 |
| **National Orthopædic Hospital for the Deformed,** 234, Gt. Portland St., W. Sec., Mr. H. Canning. Free to poor; others by letter or payment | *In-Physician*, Dr. C. Beevor. *In- and Out-Surgeons*, Messrs. F. R. Fisher, M. Th. 2; W. J. Roeckel, Tu. F. 2; E. M. Little, W. 1 |
| **Royal Orthopædic Hospital,** 297, Oxford Street, W. Sec., Mr. B. Maskell. By Governor's letter | *Surgeons*, Messrs. B. E. Brodhurst, H. A. Reeves, C. Read, W. E. Balkwill, H. F. Baker. Daily, 1 |
| **St. Bartholomew's Hospital.** Smithfield, E.C. | *Surgeon*, Mr. Walsham, M. 2.30 |
| **St. George's Hospital,** Hyde Park Corner, S.W. | W. 2. *See* General Hospitals |

### PARALYSIS, EPILEPSY, AND DISEASES OF THE NERVOUS SYSTEM GENERALLY.

| Hospitals and Terms of Admission. | Physicians, Surgeons, and Medical Officers, with Days and Hours of Attendance. |
|---|---|
| **Hospital for Epilepsy and Paralysis, and other Diseases of the Nervous System,** 32, Portland Terrace, Regent's Park Road, N.W. Sec., Mr. Howgrave Graham. By contributor's letter, or payment according to means, but free to necessitous poor | *In-Physicians*, Drs. Hughes Bennett, M.; G. Ogilvie, Tu.; J. Althaus, Th. Hour 2. *Out-Physicians*, Drs. H. Bennett, M.; G. Ogilvie, Tu.; W. H. Syers, W.; J. Althaus, Th.; Laycock, F. Hour, 2. *In- and Out-Surgeons*, Messrs. J. Bloxam, A. P. Gould, when required |

